# Underlying mechanisms and clinical potential of circRNAs in glioblastoma

**DOI:** 10.32604/or.2023.029062

**Published:** 2023-06-27

**Authors:** LEI ZHANG, YUAN ZHANG, HUIJUAN GAO, XIN LI, PEIFENG LI

**Affiliations:** Institute for Translational Medicine, The Affiliated Hospital of Qingdao University, Qingdao University, Qingdao, 266021, China

**Keywords:** CircRNAs, GBM, Underlying mechanisms, Clinical value

## Abstract

Glioblastoma (GBM) is the most malignant form of glioma and is difficult to diagnose, leading to high mortality rates. Circular RNAs (circRNAs) are noncoding RNAs with a covalently closed loop structure. CircRNAs are involved in various pathological processes and have been revealed to be important regulators of GBM pathogenesis. CircRNAs exert their biological effects by 4 different mechanisms: serving as sponges of microRNAs (miRNAs), serving as sponges of RNA binding proteins (RBPs), modulating parental gene transcription, and encoding functional proteins. Among the 4 mechanisms, sponging miRNAs is predominant. Their good stability, broad distribution and high specificity make circRNAs promising biomarkers for GBM diagnosis. In this paper, we summarized the current understanding of the characteristics and action mechanisms of circRNAs, illustrated the underlying regulatory mechanisms of circRNAs in GBM progression and explored the possible diagnostic role of circRNAs in GBM.

## Introduction

Glioblastoma is a malignant brain tumor and is also called glioblastoma multiforme (GBM) [1]. It produces many clinical symptoms such as headaches, nausea, vomiting, dystasia, speech disorders and epileptic seizures. Based on the WHO classification standard, gliomas are divided into 4 grades (I, II, III, IV) [2,3]. GBM is classified as grade IV, the most malignant grade [2,3]. GBM arises from astrocytes that support nerve cells in the brain. The five-year survival rate is less than 10% [4]. GBM develops rapidly and usually progresses to the late stage in 3–6 months. It is very difficult for doctors to treat GBM, and a complete cure is almost impossible. GBM occurs more often in older people, which further increases the difficulty of treatment. GBM has a high recurrence rate, and the overall survival rate is rather low [5,6]. Even with the best supportive care, the median survival time is less than 4 months in elderly patients [4]. With the development of clinical therapies, the median survival time has been increased to more than 15 months [7]. However, the survival time is still very short. Therefore, more efforts should be made to improve the diagnosis and prognosis and increase the median survival time of patients with GBM.

Circular RNAs (circRNAs) are noncoding RNAs and have been revealed to be closely related to tumor pathogenesis [8–10]. CircLMP2A can promote gastric carcinoma by regulating the KHSRP/VHL/HIF1α/VEGFA axis [11]. CircFoxo3 is involved in the progression of many cancers [12]. CircMAPK14 suppresses colorectal cancer progression via encoding a functional protein [13]. CircRNAs have also been revealed to regulate GBM progression [7,14]. In this paper, we summarize the classification, properties and functional mechanisms of circRNAs, explore the possible roles of circRNAs in GBM pathogenesis mainly discovered in the last three years, and elucidate the future clinical application of circRNAs in GBM diagnosis and treatment.

## Characteristics of circRNAs

CircRNAs are covalently circularized RNAs without a 5′ cap and a 3′ polyadenylated tail [15]. They are widely involved in many physiological processes and the pathogenesis of many diseases [9,16]. CircRNAs are divided into three categories according to their origin: exonic circRNAs (ecRNAs) [17], exon-intron circRNAs (EIciRNAs) [18] and circular intronic RNAs (ciRNAs) [19]. EcRNAs contain only nucleotides from exons, EIciRNAs consist of nucleotides from both exons and introns and ciRNAs contain only nucleotides from introns. The majority of circRNAs are ecRNAs. EcRNAs exist mainly in the cytoplasm, while EIciRNAs and ciRNAs exist only in the nucleus because of their intronic sequences [16].

CircRNAs have some unique strengths. As circular loops, circRNAs are very stable [20]. They have good resistance to ribonuclease (RNase) degradation, and they have higher stability than linear RNAs [21]. Moreover, circRNAs exist extensively in all living beings containing nucleated cells [22–25]. In addition, circRNA expression is highly specific in different tissues and at different developmental stages [24,26,27]. These characteristics endow them with have high diagnostic and prognostic potential.

**Figure 1:**
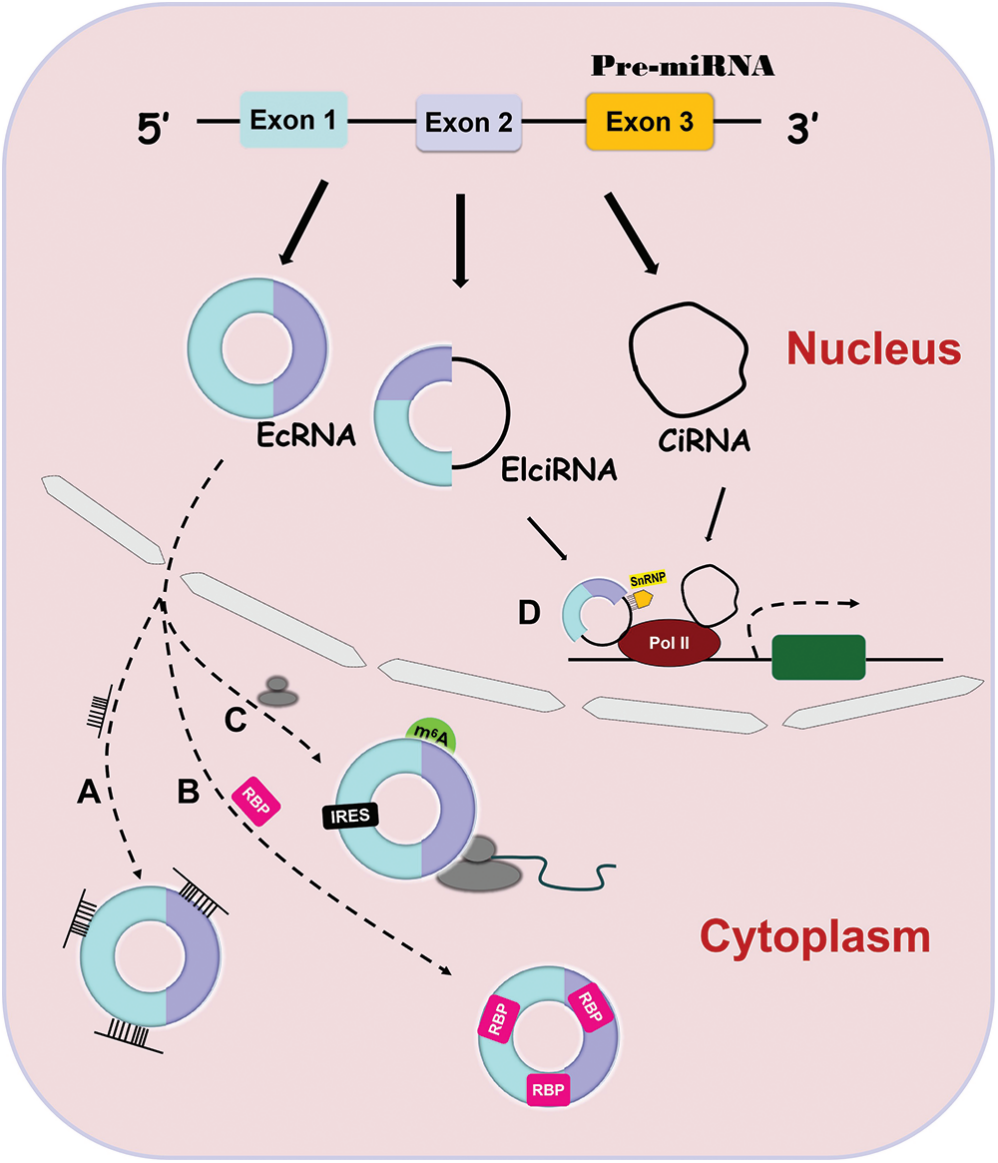
Four action mechanisms of circRNAs. CircRNAs have 4 different action mechanisms, including binding to miRNAs (Fig. 1A) or RBPs (Fig. 1B), being translation templates (Fig. 1C), or modulating parental gene expression (Fig. 1D).

CircRNAs have been demonstrated to function through different mechanisms. EcRNAs can sponge microRNAs (miRNAs) or RNA binding proteins (RBPs) to regulate the activity of downstream targets and can be translated into functional proteins to affect different pathways [15,28–31]. EcRNAs contain miRNA response elements (MREs) that promote the binding of ecRNAs to miRNAs [32] (Fig. 1A). This binding decreases miRNA expression and thus elevates the levels of the miRNA targets [32,33]. Similarly, ecRNAs can interact with RBPs to upregulate the activity of downstream targets (Fig. 1B) [34,35]. In contrast to linear RNAs, ecRNAs lack elements necessary for translation, but they can initiate protein translation via some unique elements such as the internal ribosome entry site (IRES) and N-methyladenosine (m^6^A) (Fig. 1C) [36,37]. At present, there is only one action mechanism of EIciRNAs and ciRNAs. They can bind to Polymerase II to promote parental gene transcription (Fig. 1C) [18,19].

## Functional mechanisms of circRNAs in GBM

### CircRNAs regulate GBM by interacting with miRNAs

**Figure 2:**
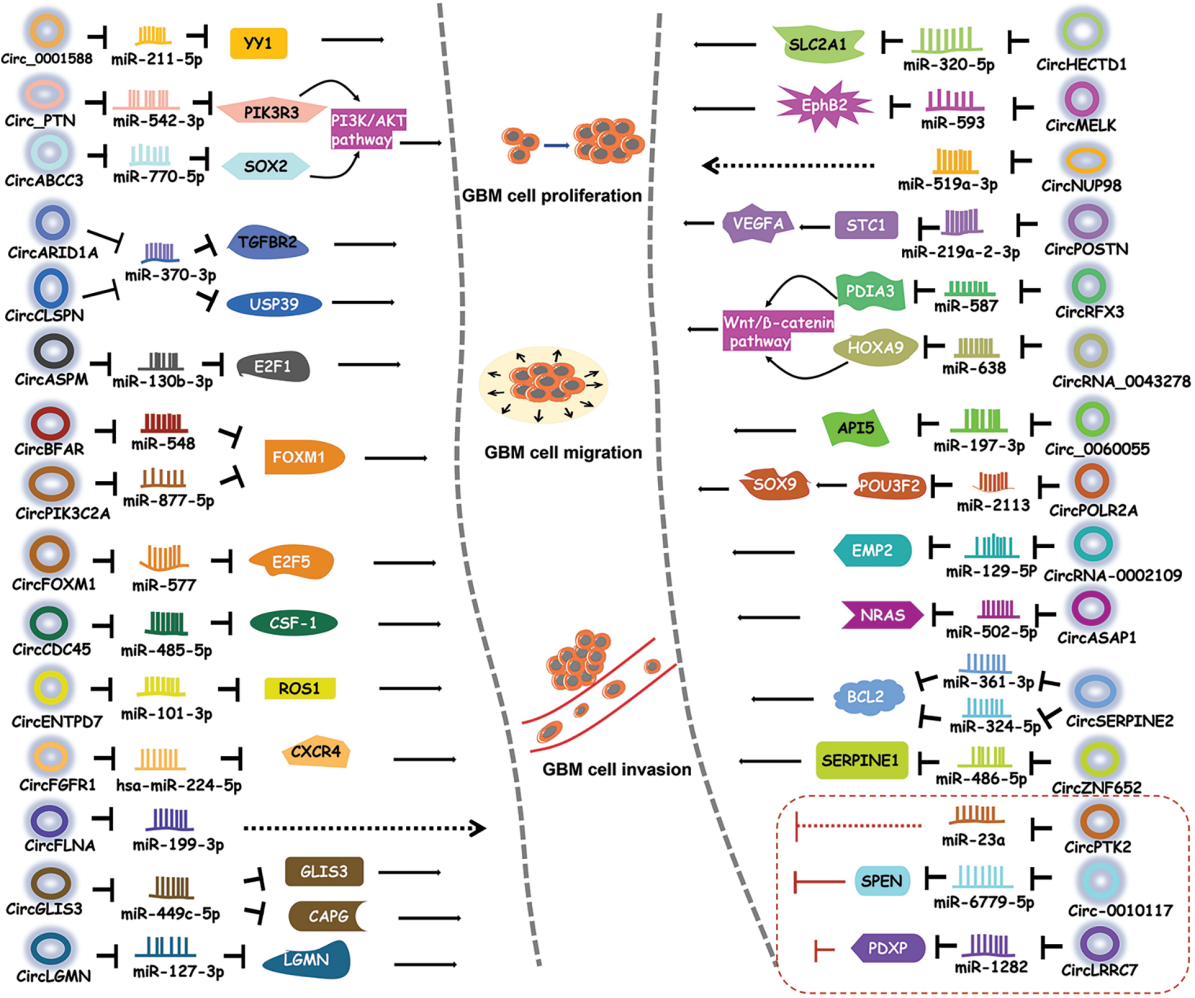
CircRNAs regulate GBM by sponging miRNAs. CircRNAs bind to miRNAs to inhibit the activities of downstream proteins, thereby participating in different signaling pathways.

Being miRNA sponge is the main mechanism of action of circRNAs [35]. A number of circRNAs have been shown to regulate GBM progression by suppressing miRNA activity [38–40] (Fig. 2).

Circ_0001588 was found to be positively correlated with poor overall survival in GBM patients [38]. Circ_0001588 knockdown inhibited GBM cell proliferation, migration and invasion and attenuated properties of GBM tumors such as tumor growth. More experiments revealed that circ_0001588 could sponge miR-211-5p to increase the activity of the Yin Yang 1 (YY1) protein, a crucial transcription factor that participates in various tumors, including GBM [41,42]. Therefore, circ_0001588 plays an oncogenic role in GBM via the miR-211-5p-YY1 axis [38].

Luo et al. [43] found that circ_PTN had obviously elevated expression in both GBM cells and cisplatin (DDP)-resistant GBM cells. DDP is a widely used antitumor drug [44,45]. Circ_PTN knockdown suppressed the DDP resistance of GBM tumors and increased the DDP sensitivity of GBM cells, while overexpression of circ_PTN enhanced DDP resistance [43]. Mechanistically, circ_PTN could bind to miR-542-3p and then upregulate the activity of the phosphoinositide-3-kinase regulatory subunit 3 (PIK3R3) protein, a receptor that activates PI3K/AKT pathway [46]. This evidence conclusively indicates that, circ_PTN elevates the DDP resistance of GBM cells by regulating the miR-542-3p-PIK3R3 pathway [43], opening an avenue for the treatment of GBM via DDP.

Li et al. [47] discovered an upregulated circular RNA, circARID1A, in GBM tissues and cells. CircARID1A knockdown inhibited GBM growth and suppressed GBM cell migration and invasion. CircARID1A was found to sponge miR-370-3p, which might target the gene encoding the transforming growth factor beta receptor 2 (TGFBR2) protein [47]. TGFBR2 is a receptor for TGF-β1 and participates in tumor migration and invasion [48]. Altogether, these observations indicate that circARID1A serves as an oncogene to promote GBM progression by binding to miR-370-3p and then upregulating TGFBR2 activity [47].

CircABCC3 was revealed to participate in GBM [49]. The expression level of circABCC3 was positively correlated with GBM stages. CircABCC3 knockdown suppressed GBM cell proliferation, migration and invasion, repressed angiogenesis and inhibited the PI3K/AKT pathway which can prevent apoptosis [49]. Mechanistic exploration demonstrated that circABCC3 could bind to miR-770-5p to upregulate SOX2 protein levels [49]. SOX2 could induce the PI3K/AKT signaling pathway. All these observations show that circABCC3 promotes GBM progression via the miR-770-5p-SOX2 axis to activate the PI3K/AKT signaling pathway [49].

CircASPM is produced from the *ASPM* gene, which has been shown to promote glioma progression [50]. Hou et al. [51] revealed that circASPM played a promoting role in GBM progression. The circASPM level was positively correlated with a poor prognosis. CircASPM knockdown inhibited GBM cell proliferation [51]. CircASPM was found to interact with miR-130b-3p and abolish its inhibitory effect on the activity of E2F transcription factor 1 (E2F1), which is related to several cancers and poor overall survival [52]. In general, all of these findings indicate that circASPM enhances GBM tumorigenesis by modulating the miR-130b-3p-E2F1 axis [51].

High circBFAR levels were found to indicate poor overall survival in GBM patients [53]. CircBFAR knockdown suppressed GBM cell proliferation and invasion. CircBFAR was identified as a sponge of miR-548b and found to inhibit the activity of miR-548b [53]. MiR-548b could target FOXM1, a transcription factor regulating the cell cycle [54]. CircBFAR knockdown elevated the expression of miR-548b and then attenuated FoxM1 activity. These results demonstrate that circBFAR might promote GBM progression through the miR-548b-FOXM1 axis [53].

CircCDC45 is an obviously highly expressed circRNA in GBM tissues and cells [55]. CircCDC45 knockdown inhibited the proliferation, invasion and migration of GBM cells and suppressed GBM tumor growth, implying the oncogenic role of circCDC45. CircCDC45 was found to sponge miR-485-5p to reduce the activity of miR-485-5p [55]. Colony-stimulating factor 1 (CSF-1) is a direct target of miR-485-5p. CSF-1 is involved in cell proliferation and invasion [56,57]. CSF-1 downregulation was found to have a similar effect as circCDC45 knockdown on GBM progression [55]. Therefore, circCDC45 could positively regulate the CSF-1 level by repressing miR-485-5p activity. In conclusion, circCDC45 plays a promoting role in GBM pathogenesis by modulating the miR-485-5p-CSF-1 pathway [55].

CircCLSPN is derived from the *CLSPN* gene that is involved in the cell cycle [58]. Downregulation of circCLSPN resulted in reductions in the viability, proliferation, invasion and migration of GBM cells and enhanced their apoptosis [59], suggesting the promoting effect of circCLSPN on GBM progression. Furthermore, miR-370-3p was validated to be the target of circCLSPN and shown to have decreased expression in GBM cells. MiR-370-3p could interact with USP39, an oncogene [60], and suppressed its activity [59]. Therefore, circCLSPN may facilitate GBM progression via the miR-370-3p-USP39 pathway.

CircENTPD7 (hsa_circ_0019421) is produced by the *ENTPD7* gene [61]. Survival analysis revealed that a high circENTPD7 level indicated a poor overall survival rate [61]. CircENTPD7 silencing suppressed GBM cell proliferation and metastasis. Further analyses determined that miR-101-3p was the target of circENTPD7 and that miR-101-3p could interact with ROS1 to inhibit its activity [61]. ROS1 is a tyrosine kinase insulin receptor that has been recognized to play a role in cancer development [62]. CircENTPD7 might bind to miR-101-3p to upregulate ROS1 activity. Overall. circENTPD7 promotes GBM by the miR-101-3p-ROS1 pathway [61].

Fibroblast growth factor receptor 1 (FGFR1) can promote cancer progression [63,64]. CircFGFR1 is derived from the *FGFR1* gene [65]. CircFGFR1 was shown to enhance GBM tumorigenesis *in vivo* and *in vitro*. Cell surface chemokine receptor (CXCR4) regulates tumor development [66,67]. The CXCR4 level was positively associated with the circFGFR1 level [65]. CXCR4 knockout eliminated the promoting effect of circFGFR1 on GBM. More experiments demonstrated that hsa-miR-224-5p could interact with both circFGFR1 and CXCR4, suggesting that circFGFR1 might sponge hsa-miR-224-5p to elevate the CXCR4 level [65]. Taken together, these findings indicate that circFGFR1 exerts an oncogenic effect on GBM by enhancing CXCR4 activity through sponging hsa-miR-224-5p [65].

CircRNA filamin A (circFLNA) is derived from the *FLNA* gene and has been found to participate in several cancers [68,69]. Sun et al. [70] revealed the role of circFLNA in GBM. Knockdown of circFLNA inhibited GBM cell proliferation and invasion. MiR-199-3p was found to be the target of circFLNA and had a negative correlation with circFLNA [70]. These results indicate that circFLNA may serve as an oncogene in GBM by sponging miR-199-3p.

Fan et al. [71] identified an upregulated circRNA in GBM tissues, circFOXM1. CirFOXM1 has been discovered to be related to the malignant development of several cancers [72,73]. CircFOXM1 silencing suppressed GBM cell growth and metastasis and GBM tumor growth [71]. CircFOXM1 could sponge miR-577 to inhibit its activity. E2F transcription factor 5 (E2F5) was revealed to be a target of miR-577 and was negatively regulated by miR-577. E2F5 regulates tumor cell proliferation and tumor growth [74,75]. E2F5 downregulation had a similar effect as circFOXM1 silencing [71]. Taken together, circFOXM1 might exacerbate GBM symptoms via targeting the miR-577-E2F5 axis.

CircGLIS3 was illustrated to be important regulator in GBM [76]. Downregulation of circGLIS3 inhibited GBM cell proliferation and induced GBM cell apoptosis [76], implying the promoting role of circGLIS3. CircGLIS3 could bind to miR-449c-5p and repress its expression [76]. Capping actin protein, gelsolin-like (CAPG) is an actin regulatory protein [77]. CAPG and GLIS3 were identified to be two targets of miR-449c-5p. CircGLIS3 positively modulated CAPG and GLIS3 levels by sponging miR-449c-5p [76]. CircGLIS3 might enhance GBM pathogenesis through the miR-449c-5p-GLIS3/CAPG axis [76].

CircHECTD1, derived from the *HECTD1* gene, has been revealed to be involved in heart diseases and some cancers [78,79]. Li et al. [80] explored the role of circHECTD1 in GBM. CircHECTD1 knockdown markedly decreased the proliferation and migration activities of GBM cells and attenuated their tumorigenicity [80]. CircHECTD1 was found to target miR-320-5p and inhibit its activity. Furthermore, miR-320-5p could interact with SLC2A1 to block its function [80]. SLC2A1 overexpression partially rescued the effect of circHECTD1 knockdown on GBM development. Overall, these results indicate that the circHECTD1-miR-320-5p-SLC2A1 axis might promote the pathogenesis of GBM [80].

Hsa_circ_0033009 (circLGMN) is derived from the mammalian legumain (*LGMN*) gene, which has been revealed to promote tumor progression [81,82]. A high circLGMN level was positively correlated with poor prognosis in GBM patients [83]. CircLGMN overexpression was found to facilitate GBM cell proliferation and invasion. CircLGMN could interact with miR-127-3p and function by sponging miR-127-2p [83]. MiR-127-3p was found to inhibit LGMN expression. Altogether, these observations demonstrate that circLGMN may aggravate GBM symptoms by suppressing miR-127-3p, thus upregulating LGMN expression [83].

Zhou et al. [84] discovered the promoting role of circMELK in glioma stem cells (GSCs) which contribute to the relapse of GBM after surgical resection. GSCs and epithelial-mesenchymal transition (EMT) promote tumor development [85]. CircMELK knockdown inhibited the progression of GBM and decreased GSC growth and migration [84], suggesting that circMELK might facilitate EMT in GBM patients and GSC maintenance. Bioinformatic analyses and experiments showed that circMELK could bind to miR-593 to repress its activity [84]. MiR-593 was then revealed to target the gene encoding the Eph receptor B2 (EphB2) protein which plays an oncogenic role in tumorigenesis [86]. CircMELK knockdown obviously decreased the EphB2 level. MiR-593 inhibitor treatment reversed the inhibitory effect of circMELK knockdown on GBM [84]. In general, these findings demonstrate that circMELK enhances GBM tumorigenesis by regulating the miR-593-EphB2 pathway.

CircNUP98 is derived from the *NUP98* gene and has been shown to be involved in renal cell carcinoma [87]. Its role in GBM was also illustrated by Lu et al. [88]. MiR-519a-3p downregulation can promote the growth of gastric cancer and hepatocellular carcinoma [89,90]. CircNUP98 was upregulated in GBM tissues, while miR-519a-3p was downregulated. Overexpression of circNUP98 facilitated GBM cell growth, whereas miR-519a-3p overexpression reduced GBM cell proliferation and viability [88]. CircNUP98 was found to bind pre-miR-519a-3p but not mature miR-519a-3p [88]. CircNUP98 might sponge pre-miR-519a-3p to block its translocation to the cytoplasm, thereby inhibiting its maturation. Therefore, circNUP98 may promote GBM tumorigenesis by suppressing the maturation of miR-519a-3p [88].

CircPIK3C2A is a highly expressed circRNA in GBM cell lines [91]. The ectopic expression of circPIK3C2A enhanced GBM cell proliferation and invasion, while circPIK3C2A knockdown decreased GBM cell proliferation and invasion [91]. CircPIK3C2A downregulation remarkably diminished tumor growth and prolonged survival in a mouse tumor model [91]. Luciferase reporter assays revealed that circPIK3C2A could sponge miR-877-5p and that miR-877-5p could target FOXM1 [91]. MiR-877-5p overexpression decreased FOXM1 expression, whereas circPIK3C2A overexpression reversed the inhibitory effect of miR-877-5p on FOXM levels [91]. These results indicate that circPIK3C2A acts as an oncogene in GBM by upregulating FOXM1 activity via sponging miR-877-5p.

CircPOSTN was found to have significantly upregulated levels in GBM tissues and cell lines [92]. CircPOSTN overexpression was found to facilitate GBM cell proliferation, migration and neovascularization, whereas circPOSTN silencing showed the opposite effects [92], suggesting a promoting role of circPOSTN in GBM. Further experiments revealed that miR-219a-2-3p was the direct target of circPOSTN and that miR-219a-2-3p could target STC1. CircPOSTN suppressed miR-219a-2-3p activity and then increased the expression of STC1 [92], thus promoting tumorigenesis. Moreover, circPOSTN was revealed to facilitate GBM neovascularization by inducing VEGFA secretion. To sum up, circPOSTN plays a tumor-promoting role in GBM via the miR-219a-2-3p-STC1-VEGFA axis [92].

CircRFX3 is generated from the *RFX3* gene [93]. A high circRFX3 expression level was related to poor prognosis in GBM patients. Overexpression of circRFX3 promoted GBM cell proliferation, invasion and migration [93]. Dual-luciferase and RNA pull-down assays revealed that circRFX3 could sponge miR-587 and that PDIA3 was a direct target of miR-587 [93]. MiR-587 inhibitor treatment partially abolished the inhibitory effect of circRFX3 knockdown on GBM development. PDIA3 knockdown inhibited the Wnt/β-catenin pathway [93]. Taken together, circRFX3 might promote GBM progression by sponging miR-587 to upregulate PDIA3 expression, and thereby regulating the Wnt/β-catenin pathway.

Circ_0060055 was found to be derived from the *EIF6* gene [40]. Circ_0060055 might play a role in mediating radioresistance in GBM. Circ_0060055 knockdown repressed the growth and invasion, promoted the apoptosis and elevated the radiosensitivity of GBM cells [40]. Bioinformatic analyses, dual-luciferase assay and RNA pull-down assay revealed the interaction between circ_0060055 and miR-197-3p. MiR-197-3p inhibitor treatment eliminated the effects of circ_0060055 silencing on GBM progression and radiosensitivity [40]. Therefore, circ_0060055 might contribute to GBM development and radioresistance by sponging miR-197-3p. Furthermore, miR-197-3p was shown to target API5 that inhibits apoptosis and is correlated with poor prognosis [94,95]. Taken together, these findings demonstrate that circ_0060055 might facilitate GBM tumorigenesis via the miR-197-3p-API5 axis [40].

Chen et al. [96] discovered an aberrantly upregulated circRNA, circPOLR2A, in GBM cells. CircPOLR2A depletion obviously inhibited the proliferation and enhanced the apoptosis of GBM cells. Further experiments found that circPOLR2A could bind to miR-2113 and suppress its inhibitory effect on POU3F2 expression [96]. POU3F2 has been reported to promote GBM development [97]. Moreover, POU3F2 was found to interact with the SOX9 promoter and activate its transcription [96]. SOX9 upregulation reversed the effect of circPOLR2A depletion on GBM cells. In conclusion, circPOLR2A contributes to GBM development via the miR-2113-POU3F2-SOX9 axis [96].

CircRNA-0002109 is generated from the *MCM10* gene, an oncogene [98]. CircRNA-0002109 downregulation diminished GBM cell proliferation, migration and invasion and suppressed tumorigenesis *in vivo*. Further mechanistic investigations revealed that circRNA-0002109 could sponge miR-19-5p to upregulate the activity of epithelial membrane protein-2 (EMP2) [98]. EMP2 is related to tumorigenesis and is positively correlated with poor prognosis [99]. MiR-19-5p overexpression attenuated the malignant phenotype of GBM cells. EMP2 overexpression reversed the inhibitory effect of circRNA-0002109 knockdown on GBM progression [98]. All these results suggest that circRNA-0002109 plays a promoting role in GBM via the miR-129-5P-EMP2 pathway.

Wei et al. [100] investigated the biological role of circASAP1 in tumorigenesis and temozolomide (TMZ) resistance in GBM patients. TMZ is an oral agent that has been used as an important part of clinical therapy for GBM clinical treatment [101]. CircASAP1 expression was increased in both GMB tissues and TMZ-resistant cells [100]. EIF4A3 participates in exon splicing and promotes circRNA generation [102]. EIF4A3 was found to increase the expression of circASAP1. Overexpression of circASAP1 facilitated GBM cell growth and TMZ resistance. CircASAP1 knockdown effectively restored TMZ sensitivity in a TMZ-resistant mouse model. Mechanistically, circASAP1 could serve as a sponge of miR-502-5p and upregulate NRAS activity by inhibiting miR-502-5p activity [100]. CircASAP1 knockdown was shown to inactivate the NRAS-MEK1/ERK1-2 signaling pathway. Therefore, these observations demonstrate that circASAP1 modulates the miR-502-5p-NRAS-MEK1/ERK1-2 signaling pathway to enhance GBM pathogenesis [100].

CircSERPINE2 has been revealed to facilitate gastric carcinoma pathogenesis [103]. Li and colleagues explored its role in GBM [104]. CircSERPINE2 had elevated expression in GBM tissues and a high circSERPINE2 level was correlated with poor overall survival in GBM patients [104]. CircSERPINE2 knockdown inhibited GBM cell proliferation and enhanced their apoptosis. CircSERPINE2 could sponge both miR-324-5p and miR-361-3p to decrease their levels. Further analyses showed that BCL2, an antiapoptotic regulator, was the direct target of both miR-324-5p and miR-362-3p [104]. More experiments revealed that circSERPINE2 could promote GBM pathogenesis by upregulating BCL2 expression via decreasing miR-361-3p/miR-324-5p levels [104].

CircZNF652 was found to have upregulated expression in GBM tissues and cell lines [105]. A high circZNF652 level indicated poor prognosis. CircZNF652 silencing abolished GBM progression *in vivo* and *in vitro* [105]. The underlying regulatory pathway was investigated. CircZNF652 could interact with miR-486-5p [105]. MiR-486-5p inhibitor treatment abolished the inhibitory effect of circZNF652 knockdown on GBM development. Furthermore, SERPINE1, a fibrinolytic inhibitor [106], was found to be targeted by miR-486-5p. CircZNF652 silencing reduced SERPINE1 expression [105]. Collectively, these findings indicate that circZNF652 aggravates GBM tumorigenesis via the miR-486-5p-SERPINE1 axis.

CircRNA_0043278 has been demonstrated to regulate the progression of several tumors [107,108]. Its role in GBM was also explored [39]. Downregulation of circ_0043278 inhibited GBM pathogenesis *in vivo* and *in vitro*. Further analysis determined that circ_0043278 directly interacted with miR-638. MiR-638 inhibitor treatment could reverse the effect of circ_0043278 knockdown [39]. MiR-638 was then revealed to interact with HOXA9, an activator of the Wnt/β-catenin signaling pathway. MiR-638 decreased HOXA9 expression, and thereby inhibited the expression of two Wnt signaling effectors, c-Myc and Cyclin D1, resulting in blockade of cell proliferation [39]. These findings conclusively show that circ_0043278 acts as an oncogene in GBM by sponging miR-638 to upregulate HOXA9 activity, thus activating the Wnt/β-catenin signaling pathway [39].

CircPTK2 can function as a tumor promoter or a tumor suppressor in different cancers [109,110]. Chen et al. [111] elaborated on the role of circPTK2 in GBM. CircPTK2 expression was reduced in GBM tissues. Overexpression of circPTK2 repressed GBM cell invasion and migration [111]. The miR-23a level was found to be inversely associated with the circPTK2 level. MiR-23a upregulation reversed the inhibitory biological effects of circPTK2 overexpression on GBM progression [111]. Taken together, these observations indicate that circPTK2 might inhibit GBM progression by decreasing miR-23a expression.

Circ-0010117 was found to be downregulated in GBM tissues [112]. Circ-0010117 depletion enhanced the proliferation and invasion of GBM cells and reduced their apoptosis, while circ-0010117 upregulation suppressed GBM tumorigenesis [112]. Circ-0010117 was found to sponge miR-6779-5p and miR-6779-5p overexpression attenuated the effect of circ-0010117 depletion on GBM. SPEN was discovered to be the direct target of miR-6779-5p and could suppress GBM progression [112]. Therefore, circ-0010117 may suppress GBM pathogenesis by upregulating SPEN by inhibiting the activity of miRNA-6779-5p.

Hsa_circ_0114014 (circLRRC7) is an ecRNA produced from the *LRRC7* gene [113]. Low circLRRC7 expression in GBM tissues was revealed to indicate poor prognosis. CircLRRC7 could repress GBM development. Analysis showed that miR-1281 might be a downstream target of circLRRC7 and that PDXP, a protein that participates in tumor metabolism [114], was the direct target of miR-1281 [113]. A high level of PDXP suggested a long overall survival time in GBM patients [113]. In summary, circLRRC7 might act as a tumor suppressor in GBM by regulating the miR-1282-PDXP pathway.

### CircRNAs regulate GBM by encoding proteins

**Figure 3:**
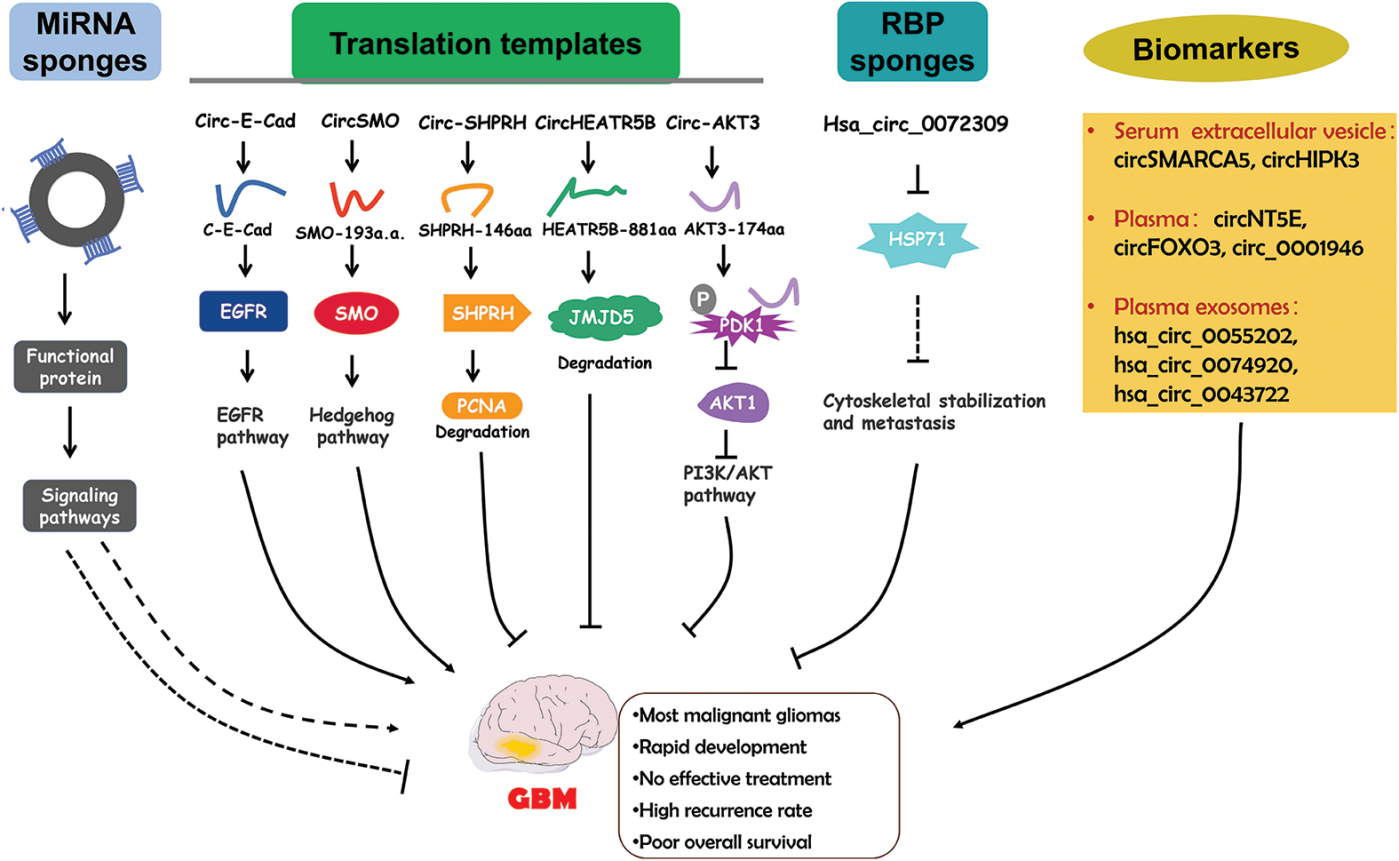
The functional roles of circRNAs in GBM and the diagnostic potential. CircRNAs regulate GBM pathogenesis via different mechanisms. Circulating circRNAs are possible biomarkers for GBM diagnosis.

Although circRNAs mainly function by serving as miRNA sponges, studies in the past five years has also discovered an increasing number of functional proteins encoded by circRNAs. CircRNA-encoded proteins have also been shown to regulate GBM progression (Fig. 3).

Circular E-cadherin (circ-E-Cad) RNA was found to be an upregulated circRNA in GBM tissues [115]. Circ-E-Cad can encode a protein in an IRES dependent manner. The encoded protein has 254 amino acids and is named circRNA-encoded E-cadherin (C-E-Cad). C-E-Cad was found to be highly expressed in GBM tissues and cells. The level of C-E-Cad was negatively correlated with prognosis in GBM patients [115]. C-E-Cad exacerbated malignant symptoms of GBM. Mechanistically, C-E-Cad could bind to the epidermal growth factor receptor (EGFR) protein to activate the EGFR signaling pathway which has been determined to promote GBM progression [115]. Altogether, circ-E-Cad can enhance GBM progression by encoding the C-E-Cad protein to stimulate the EGFR signaling pathway.

The Hedgehog (HH) pathway is important for tumorigenesis [116,117]. The G protein-coupled-like receptor smoothened (SMO) is a key factor in HH signaling [118]. CircSMO is generated from the *SMO* gene and is upregulated in GBM tissues [119]. CircSMO can be translated into a protein with 193 amino acids (referred to as SMO-193a.a.) in a manner facilitated by an IRES. A high SMO-193a.a. level was determined to indicate a low survival rate [119]. SMO-193a.a. was revealed to directly interact with SMO to induce cholesterol modification of SMO and then restore the SMO levels [119]. All these results demonstrate that circSMO serves as an oncogene by encoding a protein that regulates the HH signaling pathway.

Circ-SHPRH, derived from the SNF2 histone linker PHD RING helicase (*SHPRH*) gene, showed reduced expression in GBM tissues and cells [120]. Driven by an IRES, circ-SHPRH could be translated into the SHPRH-146aa protein. SHPRH-146aa overexpression inhibited GBM cell proliferation and tumor growth [120]. SHPRH-146aa stabilized the SHPRH protein level and then enhanced the degradation of PCNA which could induce cell proliferation [121], resulting in disrupted GBM progression [120]. Taken together, circ-SHPRH and SHPRH-146aa can suppress GBM pathogenesis.

CircHEATR5B was found to be expressed at low levels in GBM cell lines and was revealed to inhibit GBM cell proliferation [122]. CircHEATR5B can be translated into a protein with 881 amino acids (termed HEATR5B-881aa) in an IRES dependent manner [122]. HEATR5B-881aa upregulation significantly repressed glycolysis in GBM cells, thereby inhibiting GBM cell proliferation. The antitumor role of circHEATR5B in GBM was dependent on HEATR5B-881aa. Mechanistic investigation showed that HEATR5B-881aa could phosphorylate JMJD5, a protein that modulates cell metabolism, to induce its degradation [123]. Therefore, circHEATR5B and its encoded protein exert suppressive effects on GBM tumorigenesis by decreasing JMJD5 activity [122].

Circ-AKT3, derived from the *AKT3* gene, is a downregulated ecRNA in GBM tissues [124]. AKT3 is a component of an important tumor-related pathway-the RTK/PI3K/AKT pathway [125,126]. Circ-AKT3 can encode a protein containing 174 amino acids (AKT3-174aa) [124]. AKT3-174aa overexpression alleviated the malignant phenotype and reduced radioresistance. AKT3-174aa expression was shown to be positively correlated with overall survival in GBM patients [124]. The functional role of circ-AKT3 was found to be dependent on AKT3-174aa. AKT3-174aa interacted with phosphorylated PDK1 to suppress the activation of AKT. Circ-AKT3 overexpression decreased the AKT level [124]. In conclusion, AKT3-174aa functions as an antitumor regulator in GBM by regulating the PI3K/AKT pathway.

### CircRNAs regulate GBM by interacting with RBPs

Hsa_circ_0072309 was an obviously downregulated circRNA in GBM tissues [127]. Hsa_circ_0072309 overexpression abolished the proliferation and invasion of GBM cells and inhibited cytoskeletal organization in GBM cells [127]. HSP27 is a key regulator in cytoskeletal stabilization and might enhance the metastasis of tumor cells [128]. Hsa_circ_0072309 overexpression reduced the HSP27 level. HSP27 overexpression could reversed the effect of hsa_circ_0072309 overexpression on GBM development [127]. These results indicate that hsa_circ_0072309 might attenuate GBM pathogenesis by decreasing the HSP27 level (Fig. 3).

## CircRNAs as Biomarkers for GBM

Currently, the most frequently used clinical diagnostic biomarkers for cancers are proteins [129]. However, proteins are easily degraded and usually have a short half-life [130]. CircRNAs have high stability and specificity, which are important characteristics for biomarkers. Recent studies have indicated that circRNAs in the circulatory system might be valuable diagnostic biomarkers for GBM (Fig. 3).

The circSMARCA5 and circHIPK3 levels in serum extracellular vesicles (sEVs) were found to be significantly decreased in GBM patients [131]. Receiver operating characteristic curve (ROC) analysis revealed that sEV-derived circSMARCA5 and circHIPK3 had high ability to distinguish GBM patients from control subjects [131], suggesting their diagnostic potential. Furthermore, multivariate ROC analysis, performed by combining sEV-derived circSMARCA5 and circHIPK3 with known biomarkers for GBM diagnosis, resulted in improved diagnostic accuracy in GBM. All findings demonstrate that sEV-derived circSMARCA5 and circHIPK3 might be effective biomarkers for GBM diagnosis [131].

Chen et al. [132] identified three upregulated plasma circRNAs (circNT5E, circFOXO3 and circ_0001946) in GBM patients. Risk score analysis revealed that they could differentiate GBM samples from the control subject samples [132]. ROC analysis confirmed this finding and revealed that the combination of these three circRNAs had the highest ability to distinguish the GBM samples from the controls [132]. Therefore, these three circRNAs might have high potential for GBM diagnosis.

Xia et al. [133] performed a circRNA array analysis and detected three significantly upregulated circRNAs (hsa_circ_0055202, hsa_circ_0074920 and hsa_circ_0043722) in exosomes isolated from the plasma of GBM patients. ROC analysis was performed to determine their diagnostic potential. hsa_circ_0055202, hsa_circ_0074920 and hsa_circ_0043722 all had high AUCs and their combination showed the highest AUC [133], suggesting the ability of these circRNAs to predict GBM. In conclusion, these exosome-derived circRNAs (hsa_circ_0055202, hsa_circ_0074920 and hsa_circ_0043722) might be important diagnostic biomarkers for GBM patients [133].

Taken together, these studies highlight that circulating circRNAs might serve as effective biomarkers for GBM diagnosis (Table 1).

**Table 1:** Circulating circRNAs as biomarkers of GBM

Circular RNA	Source	Regulation	Clinical value	References
CircSMARCA5	Serum extracellular vesicle	Down	Diagnostic biomarker	[131]
CircHIPK3	Serum extracellular vesicle	Down	Diagnostic biomarker	[131]
CircNT5E	Plasma	Up	Diagnostic biomarker	[132]
CircFOXO3	Plasma	Up	Diagnostic biomarker	[132]
Circ_0001946	Plasma	Up	Diagnostic biomarker	[132]
Hsa_circ_0055202	Plasma exosomes	Up	Diagnostic biomarker	[133]
Hsa_circ_0074920	Plasma exosomes	Up	Diagnostic biomarker	[133]
Hsa_circ_0043722	Plasma exosomes	Up	Diagnostic biomarker	[133]

## Conclusions and Future Perspectives

All the above mentioned results show that circRNAs are important regulators of GBM pathogenesis (Table 2 and Fig. 3). The diagnosis of GBM relies on examinations in the hospital, including imaging tests and biopsy. These examinations are complicated and time-consuming and might cause damage to human patients. Therefore, innocuous and more rapid diagnostic methods are needed. CircRNAs exist in all kinds of human cells, including brain cells and GBM cells. They are highly stable compared with linear noncoding RNAs especially in circulating fluids such as plasma and serum [134]. Moreover, the process for obtaining circulating circRNAs is minimally invasive. Therefore, circRNAs are highly valuable in the diagnosis of GBM. However, the current method of circRNA detection, RT-PCR, can only be used in laboratories and still costs a long time. So it is not convenient for home use. To solve this problem, we recommend the test strip method, based on the stability of circRNAs. The test strip method has been applied for early detection of pregnancy, measurement of blood sugar, detection of influenza virus infection, and so on. During the COVID-19 pandemic, this method has also been used to detect SARS-CoV-2 antigens. The test strip method requires only a small amount of body fluid or blood, and results can be displayed in only a few seconds. Interpretation of the results is very easy and can be done at home, thereby making self-diagnosis possible. Moreover, biological probes such as gold nanocomposite probes and bioluminescence probes can be used to increase the stability of biomarkers. Therefore, the use of test strips containing circRNA-based biological probes might be an effective clinical therapy for GBM diagnosis.

**Table 2:** CircRNAs with GBM

CircRNAs	Effect	Mechanisms	Pathway	References
Circ_0001588	Promotion	Sponging miRNAs	Circ_0001588-miR-211-5p-YY1	[38]
Circ_PTN	Promotion	Sponging miRNAs	Circ_PTN-miR-542-3p-PIK3R3	[43]
CircARID1A	Promotion	Sponging miRNAs	CircARID1A-miR-370-3p-TGFBR2	[47]
CircABCC3	Promotion	Sponging miRNAs	CircABCC3-miR-770-5p-SOX2	[49]
CircASPM	Promotion	Sponging miRNAs	CircASPM-miR-130b-3p-E2F1	[51]
CircBFAR	Promotion	Sponging miRNAs	CircBFAR-miR-548b-FOXM1	[53]
CircCDC45	Promotion	Sponging miRNAs	CircCDC45-miR-485-5p-CSF-1	[55]
CircCLSPN	Promotion	Sponging miRNAs	CircCLSPN-miR-370-3p-USP39	[59]
CircENTPD7	Promotion	Sponging miRNAs	CircENTPD7-miR-101-3p-ROS1	[61]
CircFGFR1	Promotion	Sponging miRNAs	CircFGFR1-hsa-miR-224-5p-CXCR4	[65]
CircFLNA	Promotion	Sponging miRNAs	CircFLNA-miR-199-3p	[70]
CircFOXM1	Promotion	Sponging miRNAs	CircFOXM1-miR-577-E2F5	[71]
CircGLIS3	Promotion	Sponging miRNAs	CircGLIS3-miR-449c-5p-GLIS3/CAPG	[76]
CircHECTD1	Promotion	Sponging miRNAs	CircHECTD1-miR-320- 5p-SLC2A1	[80]
CircLGMN	Promotion	Sponging miRNAs	CircLGMN-miR-127-3p-LGMN	[83]
CircMELK	Promotion	Sponging miRNAs	CircMELK-miR-593-EphB2	[84]
CircNUP98	Promotion	Sponging miRNAs	CircNUP98-miR-519a-3p	[88]
CircPIK3C2A	Promotion	Sponging miRNAs	CircPIK3C2A-miR-877-5p-FOXM1	[91]
CircPOSTN	Promotion	Sponging miRNAs	CircPOSTN-miR-219a-2-3p-STC1-VEGFA	[92]
CircRFX3	Promotion	Sponging miRNAs	CircRFX3-miR-587- PDIA3-Wnt/β-catenin	[93]
Circ_0060055	Promotion	Sponging miRNAs	Circ_0060055-miR-197-3p-API5	[40]
CircPOLR2A	Promotion	Sponging miRNAs	CircPOLR2A-miR-2113-POU3F2-SOX9	[96]
circRNA-0002109	Promotion	Sponging miRNAs	CircRNA-0002109-miR-129-5P-EMP2	[98]
CircASAP1	Promotion	Sponging miRNAs	CircASAP1-miR-502-5p-NRAS	[100]
CircSERPINE2	Promotion	Sponging miRNAs	CircSERPINE2-miR-361-3p/miR-324-5p-BCL2	[104]
CircZNF652	Promotion	Sponging miRNAs	CircZNF652-miR-486-5p-SERPINE1	[105]
Circ_0043278	Promotion	Sponging miRNAs	CircRNA_0043278-miR-638-HOXA9-Wnt/ β-catenin	[39]
CircPTK2	Inhibition	Sponging miRNAs	CircPTK2-miR-23a	[111]
Circ-0010117	Inhibition	Sponging miRNAs	Circ-0010117-miR-6779-5p-SPEN	[112]
CircLRRC7	Inhibition	Sponging miRNAs	CircLRRC7-miR-1282-PDXP	[113]
Circ-E-Cad	Promotion	Encoding protein	Circ-E-Cad-C-E-Cad-EGFR	[115]
CircSMO	Promotion	Encoding protein	CircSMO-SMO-193a.a.-SMO	[119]
Circ-SHPRH	Inhibition	Encoding protein	Circ-SHPRH-SHPRH-146aa-SHPRH-PCNA	[120]
CircHEATR5B	Inhibition	Encoding protein	CircHEATR5B-HEATR5B-881aa-JMJD5	[122]
Circ-AKT3	Inhibition	Encoding protein	Circ-AKT3-AKT3-174aa-PDK1-AKT	[124]
Hsa_circ_0072309	Inhibition	Sponging RBPs	Hsa_circ_0072309-HSP27	[127]

In addition to their diagnostic value, circRNAs may also have other types of clinical potential. CircRNAs can encode functional proteins that may be more effective and convenient therapeutic targets for the development of antitumor drugs. Moreover, as demonstrated by some patents, circRNA can be used as carriers for RNAs in vaccine preparation. Currently, these applications of circRNAs have not been used in clinical practice, but these roles of circRNAs in clinical therapies are highly anticipated.

There are still several problems that should be solved before clinical application. First, based on studies from different laboratories, a single circRNA may participate in the same disease via different mechanisms. However, are there any links between these mechanisms? Which mechanism plays the main regulatory role? All these questions need further and thorough research. Second, the method for naming circRNAs is confusing. For example, one gene can give rise to more than one circRNA, all of which have the name. These circRNAs have different sequences, and their functions may also be different: circUBAP2 [135,136] and circPTK2 [109,110,137] are examples. At least two circRNAs can be generated from the *UBAP2* gene, but both are named circUBAP2 [135,136]. At least three circRNAs with different nucleic acid sequences can be produced from the *PTK2* gene, but they are all called circPTK2 [109,110,137]. Due to the sequence differences, circPTK2 performs different functions in tumors. This phenomenon causes confusion for readers and hinders follow-up research. Therefore, the method for naming circRNAs can be further improved. Third, despite the existing studies, the action mechanisms of many circulating circRNAs are still unknown or insufficiently known, constituting a hidden barrier to clinical application. More efforts should be made to address these problems.

In summary, circRNAs are widely involved in the development of GBM by suppressing the activities of downstream targets (miRNAs/RBPs) or encoding functional proteins. All these findings provide new ideas for the prevention and treatment of GBM. Although studies have demonstrated that circRNAs have high clinical value, their clinical implementation still has a long way to go. How to solve the problems from basic research to clinical application will be a key point of future research. We have great confidence in the clinical actualization of circRNAs in GBM.

## References

[1] McKinnon, C., Nandhabalan, M., Murray, S. A., Plaha, P. (2021). Glioblastoma: Clinical presentation, diagnosis, and management. British Medical Journal*,* 374*,* n1560. 10.1136/bmj.n1560; 34261630

[2] Broekman, M. L., Maas, S. L. N., Abels, E. R., Mempel, T. R., Krichevsky, A. M. et al. (2018). Multidimensional communication in the microenvirons of glioblastoma. Nature Reviews Neurology*,* 14 *(*8*),* 482–495. 10.1038/s41582-018-0025-8; 29985475 PMC6425928

[3] Caragher, S. P., Hall, R. R., Ahsan, R., Ahmed, A. U. (2018). Monoamines in glioblastoma: Complex biology with therapeutic potential. Neuro-Oncology*,* 20 *(*8*),* 1014–1025. 10.1093/neuonc/nox210; 29126252 PMC6280144

[4] Tan, A. C., Ashley, D. M., Lopez, G. Y., Malinzak, M., Friedman, H. S. et al. (2020). Management of glioblastoma: State of the art and future directions. CA: A Cancer Journal for Clinicians*,* 70 *(*4*),* 299–312. 10.3322/caac.21613; 32478924

[5] Balca-Silva, J., Matias, D., Carmo, A. D., Sarmento-Ribeiro, A. B., Lopes, M. C. et al. (2019). Cellular and molecular mechanisms of glioblastoma malignancy: Implications in resistance and therapeutic strategies. Seminars in Cancer Biology*,* 58 *(*90–105*),* 130–141. 10.1016/j.semcancer.2018.09.007; 30266571

[6] Lim, M., Xia, Y., Bettegowda, C., Weller, M. (2018). Current state of immunotherapy for glioblastoma. Nature Reviews Clinical Oncology*,* 15 *(*7*),* 422–442. 10.1038/s41571-018-0003-5; 29643471

[7] Guo, X., Piao, H. (2021). Research progress of circRNAs in glioblastoma. Frontiers in Cell and Developmental Biology*,* 9*,* 791892. 10.3389/fcell.2021.791892; 34881248 PMC8645988

[8] Wang, M., Yu, F., Zhang, Y., Zhang, L., Chang, W. et al. (2022). The emerging roles of circular RNAs in the chemoresistance of gastrointestinal cancer. Frontiers in Cell and Developmental Biology*,* 10*,* 821609. 10.3389/fcell.2022.821609; 35127685 PMC8814461

[9] Zhou, X., Ao, X., Jia, Z., Li, Y., Kuang, S. et al. (2022). Non-coding RNA in cancer drug resistance: Underlying mechanisms and clinical applications. Frontiers in Oncology*,* 12*,* 951864. 10.3389/fonc.2022.951864; 36059609 PMC9428469

[10] Ao, X., Ding, W., Li, X., Xu, Q., Chen, X. et al. (2023). Non-coding RNAs regulating mitochondrial function in cardiovascular diseases. Journal of Molecular Medicine*,* 19 *(*304–308*),* 59. 10.1007/s00109-023-02305-8; 37014377

[11] Du, Y., Zhang, J. Y., Gong, L. P., Feng, Z. Y., Wang, D. et al. (2022). Hypoxia-induced ebv-circLMP2A promotes angiogenesis in EBV-associated gastric carcinoma through the KHSRP/VHL/HIF1alpha/VEGFA pathway. Cancer Letters*,* 526*,* 259–272. 10.1016/j.canlet.2021.11.031; 34863886

[12] Zhang, L., Wang, Y., Zhang, Y., Zhao, Y., Li, P. (2021). Pathogenic mechanisms and the potential clinical value of circFoxo3 in cancers. Molecular Therapy-Nucleic Acids*,* 23 *(*Suppl 6*),* 908–917. 10.1016/j.omtn.2021.01.010; 33614239 PMC7868936

[13] Wang, L., Zhou, J., Zhang, C., Chen, R., Sun, Q. et al. (2021). A novel tumour suppressor protein encoded by circMAPK14 inhibits progression and metastasis of colorectal cancer by competitively binding to MKK6. Clinical and Translational Medicine*,* 11 *(*10*),* e613. 10.1002/ctm2.613; 34709743 PMC8516360

[14] Sun, J., Li, B., Shu, C., Ma, Q., Wang, J. (2020). Functions and clinical significance of circular RNAs in glioma. Molecular Cancer*,* 19 *(*1*),* 34. 10.1186/s12943-019-1121-0; 32061256 PMC7023692

[15] Ao, X., Liu, Y. (2022). Novel insights into circular RNA regulation in arsenic-exposure-induced lung cancer. Molecular Therapy-Oncolytics*,* 27*,* 200–202. 10.1016/j.omto.2022.10.010; 36381655 PMC9661420

[16] Memczak, S., Jens, M., Elefsinioti, A., Torti, F., Krueger, J. et al. (2013). Circular RNAs are a large class of animal RNAs with regulatory potency. Nature*,* 495 *(*7441*),* 333–338. 10.1038/nature11928; 23446348

[17] Zhang, X. O., Wang, H. B., Zhang, Y., Lu, X., Chen, L. L. et al. (2014). Complementary sequence-mediated exon circularization. Cell*,* 159 *(*1*),* 134–147. 10.1016/j.cell.2014.09.001; 25242744

[18] Li, Z., Huang, C., Bao, C., Chen, L., Lin, M. et al. (2015). Exon-intron circular RNAs regulate transcription in the nucleus. Nature Structural & Molecular Biology*,* 22 *(*3*),* 256–264. 10.1038/nsmb.2959; 25664725

[19] Zhang, Y., Zhang, X. O., Chen, T., Xiang, J. F., Yin, Q. F. et al. (2013). Circular intronic long noncoding RNAs. Molecular Cell*,* 51 *(*6*),* 792–806. 10.1016/j.molcel.2013.08.017; 24035497

[20] Zhang, L., Zhang, Y., Wang, Y., Zhao, Y., Ding, H. et al. (2020). Circular RNAs: Functions and clinical significance in cardiovascular disease. Frontiers in Cell and Developmental Biology*,* 8*,* 584051. 10.3389/fcell.2020.584051; 33134301 PMC7550538

[21] Suzuki, H., Zuo, Y. H., Wang, J. H., Zhang, M. Q., Malhotra, A. et al. (2006). Characterization of RNase R-digested cellular RNA source that consists of lariat and circular RNAs from pre-mRNA splicing. Nucleic Acids Research*,* 34 *(*8*),* e63. 10.1093/nar/gkl151; 16682442 PMC1458517

[22] Jeck, W. R., Sorrentino, J. A., Wang, K., Slevin, M. K., Burd, C. E. et al. (2013). Circular RNAs are abundant, conserved, and associated with ALU repeats. RNA*,* 19 *(*2*),* 141–157. 10.1261/rna.035667.112; 23249747 PMC3543092

[23] Zheng, Q. P., Bao, C. Y., Guo, W. J., Li, S. Y., Chen, J. et al. (2016). Circular RNA profiling reveals an abundant circHIPK3 that regulates cell growth by sponging multiple miRNAs. Nature Communications*,* 7 *(*1*),* 11215. 10.1038/ncomms11215; 27050392 PMC4823868

[24] Xu, T. Y., Wu, J., Han, P., Zhao, Z. M., Song, X. F. (2017). Circular RNA expression profiles and features in human tissues: A study using RNA-seq data. BMC Genomics*,* 18 *(*S6*),* 680. 10.1186/s12864-017-4029-3; 28984197 PMC5629547

[25] Zeng, X., Lin, W., Guo, M., Zou, Q. (2017). A comprehensive overview and evaluation of circular RNA detection tools. PLoS Computational Biology*,* 13 *(*6*),* e1005420. 10.1371/journal.pcbi.1005420; 28594838 PMC5466358

[26] Jakobi, T., Czaja-Hasse, L. F., Reinhardt, R., Dieterich, C. (2016). Profiling and validation of the circular RNA repertoire in adult murine hearts. Genomics Proteomics & Bioinformatics*,* 14 *(*4*),* 216–223. 10.1016/j.gpb.2016.02.003; 27132142 PMC4996846

[27] Li, Y. S., Zhang, J. W., Huo, C. Q., Ding, N., Li, J. Y. et al. (2017). Dynamic organization of lncRNA and circular RNA regulators collectively controlled cardiac differentiation in humans. Ebiomedicine*,* 24 *(*5*),* 137–146. 10.1016/j.ebiom.2017.09.015; 29037607 PMC5652025

[28] Wang, S., Chen, J. Y., Yu, W. Q., Deng, F. (2018). Circular RNA DLGAP4 ameliorates cardiomyocyte apoptosis through regulating BCL2 via targeting miR-143 in myocardial ischemia-reperfusion injury. International Journal of Cardiology*,* 279 *(*1*),* 147. 10.1016/j.ijcard.2018.09.023; 30213603

[29] Kong, Z., Wan, X. C., Lu, Y. L., Zhang, Y. Y., Huang, Y. et al. (2020). Circular RNA circFOXO3 promotes prostate cancer progression through sponging miR-29a-3p. Journal of Cellular and Molecular Medicine*,* 24 *(*1*),* 799–813. 10.1111/jcmm.14791; 31733095 PMC6933405

[30] Abdelmohsen, K., Panda, A. C., Munk, R., Grammatikakis, I., Dudekula, D. B. et al. (2017). Identification of HuR target circular RNAs uncovers suppression of PABPN1 translation by CircPABPN1. RNA Biology*,* 14 *(*3*),* 361–369. 10.1080/15476286.2017.1279788; 28080204 PMC5367248

[31] Yang, Z. G., Awan, F. M., Du, W. W., Zeng, Y., Lyu, J. et al. (2017). The circular RNA interacts with STAT3, Increasing its nuclear translocation and wound repair by modulating Dnmt3a and miR-17 function. Molecular Therapy*,* 25 *(*9*),* 2062–2074. 10.1016/j.ymthe.2017.05.022; 28676341 PMC5589065

[32] Hansen, T. B., Jensen, T. I., Clausen, B. H., Bramsen, J. B., Finsen, B. et al. (2013). Natural RNA circles function as efficient microRNA sponges. Nature*,* 495 *(*7441*),* 384–388. 10.1038/nature11993; 23446346

[33] Garikipati, V. N. S., Verma, S. K., Cheng, Z. J., Liang, D. M., Truongcao, M. M. et al. (2019). Circular RNA circFndc3b modulates cardiac repair after myocardial infarction via FUS/VEGF-A axis. Nature Communications*,* 10*,* 4317. 10.1038/s41467-019-11777-7; 31541092 PMC6754461

[34] Du, W. W., Yang, W., Liu, E., Yang, Z., Dhaliwal, P. et al. (2016). Foxo3 circular RNA retards cell cycle progression via forming ternary complexes with p21 and CDK2. Nucleic Acids Research*,* 44 *(*6*),* 2846–2858. 10.1093/nar/gkw027; 26861625 PMC4824104

[35] Zhang, L., Zhang, Y., Yu, F., Li, X., Gao, H. et al. (2022). The circRNA-miRNA/RBP regulatory network in myocardial infarction. Frontiers in Pharmacology*,* 13*,* 941123. 10.3389/fphar.2022.941123; 35924059 PMC9340152

[36] Pamudurti, N. R., Bartok, O., Jens, M., Ashwal-Fluss, R., Stottmeister, C. et al. (2017). Translation of circRNAs. Molecular Cell*,* 66 *(*1*),* 9–21.e7. 10.1016/j.molcel.2017.02.021; 28344080 PMC5387669

[37] Yang, Y., Fan, X., Mao, M., Song, X., Wu, P. et al. (2017). Extensive translation of circular RNAs driven by *N*^6^-methyladenosine. Cell Research*,* 27 *(*5*),* 626–641. 10.1038/cr.2017.31; 28281539 PMC5520850

[38] Wang, Q., Zheng, D., Li, Y., Zhang, Y., Sui, R. et al. (2021). Circular RNA circ_0001588 sponges miR-211-5p to facilitate the progression of glioblastoma via up-regulating YY1 expression. Journal of Gene Medicine*,* 23*,* e3371. 10.1002/jgm.3371; 34105224

[39] Wu, Z., Zheng, M., Zhang, Y., Xie, M., Tian, S. et al. (2020). Hsa_circ_0043278 functions as competitive endogenous RNA to enhance glioblastoma multiforme progression by sponging miR-638. Aging*,* 12 *(*21*),* 21114–21128. 10.18632/aging.103603; 33154193 PMC7695414

[40] Yuan, J., Liu, Z., Liu, J., Fan, R. (2022). Circ_0060055 promotes the growth, invasion, and radioresistance of glioblastoma by targeting miR-197-3p/API5 Axis. Neurotoxicity Research*,* 40 *(*5*),* 1292–1303. 10.1007/s12640-022-00548-w; 35849320

[41] Jia, B., Liu, W., Gu, J., Wang, J., Lv, W. et al. (2019). MiR-7-5p suppresses stemness and enhances temozolomide sensitivity of drug-resistant glioblastoma cells by targeting Yin Yang 1. Experimental Cell Research*,* 375 *(*1*),* 73–81. 10.1016/j.yexcr.2018.12.016; 30586549

[42] Wu, S., Wang, H., Li, Y., Xie, Y., Huang, C. et al. (2018). Transcription factor YY1 promotes cell proliferation by directly activating the pentose phosphate pathway. Cancer Research*,* 78*,* 4549–4562. 10.1158/0008-5472.CAN-17-4047; 29921695

[43] Luo, H., Yi, T., Huang, D., Chen, X., Li, X. et al. (2021). circ_PTN contributes to -cisplatin resistance in glioblastoma via PI3K/AKT signaling through the miR-542-3p/PIK3R3 pathway. Molecular Therapy-Nucleic Acids*,* 26*,* 1255–1269. 10.1016/j.omtn.2021.08.034; 34853725 PMC8607136

[44] Hu, Y., Zhu, Q. N., Deng, J. L., Li, Z. X., Wang, G. et al. (2018). Emerging role of long non-coding RNAs in cisplatin resistance. OncoTargets and Therapy*,* 11*,* 3185–3194. 10.2147/OTT 29881292 PMC5983019

[45] Liu, Y., Ao, X., Jia, Y., Li, X., Wang, Y. et al. (2022). The FOXO family of transcription factors: Key molecular players in gastric cancer. Journal of Molecular Medicine*,* 100 *(*7*),* 997–1015. 10.1007/s00109-022-02219-x; 35680690

[46] Zhou, J., Chen, G. B., Tang, Y. C., Sinha, R. A., Wu, Y. et al. (2012). Genetic and bioinformatic analyses of the expression and function of PI3K regulatory subunit PIK3R3 in an Asian patient gastric cancer library. BMC Medical Genomics*,* 5 *(*1*),* 34. 10.1186/1755-8794-5-34; 22876838 PMC3479415

[47] Li, B., Chen, J., Wu, Y., Luo, H., Ke, Y. (2022). Decrease of circARID1A retards glioblastoma invasion by modulating miR-370-3p/TGFBR2 pathway. International Journal of Biological Sciences*,* 18 *(*13*),* 5123–5135. 10.7150/ijbs.66673; 35982888 PMC9379412

[48] Wang, Q., Cai, J., Fang, C., Yang, C., Zhou, J. et al. (2018). Mesenchymal glioblastoma constitutes a major ceRNA signature in the TGF-β pathway. Theranostics*,* 8 *(*17*),* 4733–4749. 10.7150/thno.26550; 30279734 PMC6160778

[49] Zhang, H., Xu, W. (2021). CircABCC3 knockdown inhibits glioblastoma cell malignancy by regulating miR-770-5p/SOX2 axis through PI3K/AKT signaling pathway. Brain Research*,* 1764 *(*2*),* 147465. 10.1016/j.brainres.2021.147465; 33811842

[50] Chen, X., Huang, L., Yang, Y., Chen, S., Sun, J. et al. (2020). ASPM promotes glioblastoma growth by regulating G1 restriction point progression and Wnt-beta-catenin signaling. Aging*,* 12 *(*1*),* 224–241. 10.18632/aging.102612; 31905171 PMC6977704

[51] Hou, D., Wang, Z., Li, H., Liu, J., Liu, Y. et al. (2022). Circular RNA circASPM promotes the progression of glioblastoma by acting as a competing endogenous RNA to regulate miR-130b-3p/E2F1 axis. Journal of Cancer*,* 13 *(*5*),* 1664–1678. 10.7150/jca.57691; 35371308 PMC8965134

[52] DeGregori, J., Kowalik, T., Nevins, J. R. (1995). Cellular targets for activation by the E2F1 transcription factor include DNA synthesis- and G1/S-regulatory genes. Molecular and Cellular Biology*,* 15 *(*8*),* 4215–4224. 10.1128/MCB.15.8.4215; 7623816 PMC230660

[53] Li, C., Guan, X., Jing, H., Xiao, X., Jin, H. et al. (2022). Circular RNA circBFAR promotes glioblastoma progression by regulating a miR-548b/FoxM1 axis. FASEB Journal*,* 36 *(*3*),* e22183. 10.1096/fj.202101307R; 35202487

[54] Kwok, C. T. D., Leung, M. H., Qin, J., Qin, Y., Wang, J. et al. (2016). The Forkhead box transcription factor FOXM1 is required for the maintenance of cell proliferation and protection against oxidative stress in human embryonic stem cells. Stem Cell Research*,* 16 *(*3*),* 651–661. 10.1016/j.scr.2016.03.007; 27062359

[55] Liu, R., Dai, W., Wu, A., Li, Y. (2021). CircCDC45 promotes the malignant progression of glioblastoma by modulating the miR-485-5p/CSF-1 axis. BMC Cancer*,* 21 *(*1*),* 1090. 10.1186/s12885-021-08803-7; 34627193 PMC8501713

[56] De, I., Steffen, M. D., Clark, P. A., Patros, C. J., Sokn, E. et al. (2016). CSF1 overexpression promotes high-grade glioma formation without impacting the polarization status of glioma-associated microglia and macrophages. Cancer Research*,* 76*,* 2552–2560. 10.1158/0008-5472.CAN-15-2386; 27013192 PMC4873447

[57] Stafford, J. H., Hirai, T., Deng, L., Chernikova, S. B., Urata, K. et al. (2016). Colony stimulating factor 1 receptor inhibition delays recurrence of glioblastoma after radiation by altering myeloid cell recruitment and polarization. Neuro-Oncology*,* 18 *(*6*),* 797–806. 10.1093/neuonc/nov272; 26538619 PMC4864255

[58] Smits, V. A. J., Cabrera, E., Freire, R., Gillespie, D. A. (2019). Claspin-checkpoint adaptor and DNA replication factor. FEBS Journal*,* 286 *(*3*),* 441–455. 10.1111/febs.14594; 29931808

[59] Hu, T., Lei, D., Zhou, J., Zhang, B. O. (2021). circRNA derived from CLSPN (circCLSPN) is an oncogene in human glioblastoma multiforme by regulating cell growth, migration and invasion via ceRNA pathway. Journal of Biosciences*,* 46 *(*3*),* 66. 10.1007/s12038-021-00185-z 34269180

[60] Ding, K., Ji, J., Zhang, X., Huang, B., Chen, A. et al. (2019). RNA splicing factor USP39 promotes glioma progression by inducing TAZ mRNA maturation. Oncogene*,* 38 *(*37*),* 6414–6428. 10.1038/s41388-019-0888-1; 31332287 PMC6756117

[61] Zhu, F., Cheng, C., Qin, H., Wang, H., Yu, H. (2020). A novel circular RNA circENTPD7 contributes to glioblastoma progression by targeting ROS1. Cancer Cell International*,* 20 *(*1*),* 118. 10.1186/s12935-020-01208-9; 32308563 PMC7147020

[62] Kris, M. G., Johnson, B. E., Berry, L. D., Kwiatkowski, D. J., Iafrate, A. J. et al. (2014). Using multiplexed assays of oncogenic drivers in lung cancers to select targeted drugs. Journal of the American Medical Association*,* 311 *(*19*),* 1998–2006. 10.1001/jama.2014.3741; 24846037 PMC4163053

[63] Kong, S., Cao, Y., Li, X., Li, Z., Xin, Y. et al. (2020). MiR-3116 sensitizes glioma cells to temozolomide by targeting FGFR1 and regulating the FGFR1/PI3K/AKT pathway. Journal of Cellular and Molecular Medicine*,* 24 *(*8*),* 4677–4686. 10.1111/jcmm.15133; 32181582 PMC7176860

[64] Zhang, P. F., Pei, X., Li, K. S., Jin, L. N., Wang, F. et al. (2019). Circular RNA circFGFR1 promotes progression and anti-PD-1 resistance by sponging miR-381-3p in non-small cell lung cancer cells. Molecular Cancer*,* 18 *(*1*),* 179. 10.1186/s12943-019-1111-2; 31815619 PMC6900862

[65] Zhang, Q., Chen, S., Zhen, Y., Gao, P., Zhang, Z. et al. (2022). Circular RNA circFGFR1 functions as an oncogene in glioblastoma cells through sponging to hsa-miR-224-5p. Journal of Immunology Research*,* 2022*,* 1–14. 10.1155/2022/7990251; 35059468 PMC8764274

[66] Gagliardi, F., Narayanan, A., Reni, M., Franzin, A., Mazza, E. et al. (2014). The role of CXCR4 in highly malignant human gliomas biology: Current knowledge and future directions. Glia*,* 62 *(*7*),* 1015–1023. 10.1002/glia.22669; 24715652

[67] Guo, F., Wang, Y., Liu, J., Mok, S. C., Xue, F. et al. (2016). CXCL12/CXCR4: A symbiotic bridge linking cancer cells and their stromal neighbors in oncogenic communication networks. Oncogene*,* 35 *(*7*),* 816–826. 10.1038/onc.2015.139; 25961926

[68] Qu, J., Yang, J., Chen, M., Wei, R., Tian, J. (2020). CircFLNA acts as a sponge of miR-646 to facilitate the proliferation, metastasis, glycolysis, and apoptosis inhibition of gastric cancer by targeting PFKFB2. Cancer Management and Research*,* 12*,* 8093–8103. 10.2147/CMAR.S264674; 32982406 PMC7490063

[69] Wang, J. X., Liu, Y., Jia, X. J., Liu, S. X., Dong, J. H. et al. (2019). Upregulation of circFLNA contributes to laryngeal squamous cell carcinoma migration by circFLNA-miR-486-3p-FLNA axis. Cancer Cell International*,* 19 *(*1*),* 196. 10.1186/s12935-019-0924-9; 31384171 PMC6664525

[70] Sun, Y., Ma, G., Xiang, H., Wang, X., Wang, H. et al. (2021). circFLNA promotes glioblastoma proliferation and invasion by negatively regulating miR-199-3p expression. Molecular Medicine Reports*,* 24 *(*5*),* 786. 10.3892/mmr.2021.12426; 34498720 PMC8441964

[71] Fan, X., Liu, M., Fei, L., Huang, Z., Yan, Y. (2022). CircFOXM1 promotes the proliferation, migration, invasion, and glutaminolysis of glioblastoma by regulating the miR-577/E2F5 axis. Bosnian Journal of Basic Medical Sciences*,* 22*,* 205–216. 10.17305/bjbms.2021.6028; 34784267 PMC8977084

[72] Wang, G., Jiang, Y., Lu, C., Jiang, W., Wu, S. et al. (2021). CircFOXM1 promotes proliferation and metastasis of hepatocellular carcinoma via regulating miR-1179/SPAG5 axis. Scientific Reports*,* 11 *(*1*),* 23890. 10.1038/s41598-021-03285-w; 34903799 PMC8668908

[73] Ye, M., Hou, H., Shen, M., Dong, S., Zhang, T. (2020). Circular RNA circFOXM1 plays a role in papillary thyroid carcinoma by sponging miR-1179 and regulating HMGB1 expression. Molecular Therapy-Nucleic Acids*,* 19*,* 741–750. 10.1016/j.omtn.2019.12.014; 31951855 PMC6965511

[74] Fang, D. Z., Wang, Y. P., Liu, J., Hui, X. B., Wang, X. D. et al. (2018). MicroRNA-129-3p suppresses tumor growth by targeting E2F5 in glioblastoma. European Review for Medical and Pharmacological Sciences*,* 22*,* 1044–1050. 10.26355/eurrev_201802_14387; 29509253

[75] Xu, X., Cai, N., Zhi, T., Bao, Z., Wang, D. et al. (2017). MicroRNA-1179 inhibits glioblastoma cell proliferation and cell cycle progression via directly targeting E2F transcription factor 5. American Journal of Cancer Research*,* 7*,* 1680–1692; 28861324 PMC5574940

[76] Zhou, Q., Shaya, M., Kugeluke, Y., Fu, Q., Li, S. et al. (2022). A circular RNA derived from GLIS3 accelerates the proliferation of glioblastoma cells through competitively binding with miR-449c-5p to upregulate CAPG and GLIS3. BMC Neuroscience*,* 23 *(*1*),* 53. 10.1186/s12868-022-00736-6; 36114444 PMC9479268

[77] Yun, D. P., Wang, Y. Q., Meng, D. L., Ji, Y. Y., Chen, J. X. et al. (2018). Actin-capping protein CapG is associated with prognosis, proliferation and metastasis in human glioma. Oncology Reports*,* 39*,* 1011–1022. 10.3892/or.2018.6225; 29399702 PMC5802022

[78] Cai, J., Chen, Z., Wang, J., Wang, J., Chen, X. et al. (2019). circHECTD1 facilitates glutaminolysis to promote gastric cancer progression by targeting miR-1256 and activating beta-catenin/c-Myc signaling. Cell Death & Disease*,* 10 *(*8*),* 576. 10.1038/s41419-019-1814-8; 31371702 PMC6675787

[79] Jiang, Q. L., Feng, S. J., Yang, Z. Y., Xu, Q., Wang, S. Z. (2020). CircHECTD1 up-regulates mucin 1 expression to accelerate hepatocellular carcinoma development by targeting microRNA-485-5p via a competing endogenous RNA mechanism. Chinese Medical Journal*,* 133 *(*15*),* 1774–1785. 10.1097/CM9.0000000000000917; 32675746 PMC7469999

[80] Li, W., Wang, S., Shan, B., Cheng, X., He, H. et al. (2021). CircHECTD1 regulates cell proliferation and migration by the mir-320-5p/SLC2A1 axis in glioblastoma multiform. Frontiers in Oncology*,* 11*,* 666391. 10.3389/fonc.2021.666391; 34079759 PMC8166227

[81] Lin, Y., Qiu, Y., Xu, C., Liu, Q., Peng, B. et al. (2014). Functional role of asparaginyl endopeptidase ubiquitination by TRAF6 in tumor invasion and metastasis. Journal of the National Cancer Institute*,* 106 *(*4*),* dju012. 10.1093/jnci/dju012; 24610907

[82] Zhang, W., Lin, Y. (2021). The mechanism of asparagine endopeptidase in the progression of malignant tumors: A review. Cells*,* 10 *(*5*),* 1153. 10.3390/cells10051153; 34068767 PMC8151911

[83] Chen, B., Wang, M., Huang, R., Liao, K., Wang, T. et al. (2021). Circular RNA circLGMN facilitates glioblastoma progression by targeting miR-127-3p/LGMN axis. Cancer Letters*,* 522*,* 225–237. 10.1016/j.canlet.2021.09.030; 34582975

[84] Zhou, F., Wang, B., Wang, H., Hu, L., Zhang, J. et al. (2021). circMELK promotes glioblastoma multiforme cell tumorigenesis through the miR-593/EphB2 axis. Molecular Therapy-Nucleic Acids*,* 25 *(*Suppl 1*),* 25–36. 10.1016/j.omtn.2021.05.002; 34168916 PMC8190146

[85] Chen, J., McKay, R. M., Parada, L. F. (2012). Malignant glioma: Lessons from genomics, mouse models, and stem cells. Cell*,* 149 *(*1*),* 36–47. 10.1016/j.cell.2012.03.009; 22464322 PMC3719882

[86] Wang, S. D., Rath, P., Lal, B., Richard, J. P., Li, Y. et al. (2012). EphB2 receptor controls proliferation/migration dichotomy of glioblastoma by interacting with focal adhesion kinase. Oncogene*,* 31 *(*50*),* 5132–5143. 10.1038/onc.2012.16; 22310282 PMC3349801

[87] Yu, R., Yao, J., Ren, Y. (2020). A novel circRNA, circNUP98, a potential biomarker, acted as an oncogene via the miR-567/PRDX3 axis in renal cell carcinoma. Journal of Cellular and Molecular Medicine*,* 24 *(*17*),* 10177–10188. 10.1111/jcmm.15629; 32729669 PMC7520319

[88] Lu, J., Lou, G., Jiang, L., Liu, X., Jiang, J. et al. (2021). CircNUP98 suppresses the maturation of mir-519a-3p in glioblastoma. Frontiers in Neurology*,* 12*,* 679745. 10.3389/fneur.2021.679745; 34867700 PMC8636316

[89] Cai, H., Lin, H., Cao, W., Sun, J., Huang, Y. et al. (2019). The downregulation of miR-519a predicts poor prognosis and contributes to tumor progression in gastric cancer. International Journal of Clinical and Experimental Pathology*,* 12*,* 2496–2505; 31934076 PMC6949548

[90] Tu, K., Liu, Z., Yao, B., Han, S., Yang, W. et al. (2016). MicroRNA-519a promotes tumor growth by targeting PTEN/PI3K/AKT signaling in hepatocellular carcinoma. International Journal of Oncology*,* 48 *(*3*),* 965–974. 10.3892/ijo.2015.3309; 26708293 PMC4750540

[91] Yang, J., Tian, S., Wang, B., Wang, J., Cao, L. et al. (2021). CircPIK3C2A facilitates the progression of glioblastoma via targeting mir-877-5p/FOXM1 Axis. Frontiers in Oncology*,* 11*,* 801776. 10.3389/fonc.2021.801776; 35004326 PMC8739489

[92] Long, N., Xu, X., Lin, H., Lv, Y., Zou, S. et al. (2022). Circular RNA circPOSTN promotes neovascularization by regulating miR-219a-2-3p/STC1 axis and stimulating the secretion of VEGFA in glioblastoma. Cell Death Discovery*,* 8 *(*1*),* 349. 10.1038/s41420-022-01136-9; 35927233 PMC9352789

[93] Li, T., Xu, J., Liu, Y. (2021). A novel circular RNA CircRFX3 serves as a sponge for microRNA-587 in promoting glioblastoma progression via regulating PDIA3. Frontiers in Cell and Developmental Biology*,* 9*,* 757260. 10.3389/fcell.2021.757260; 34950658 PMC8691731

[94] Bousquet, G., Feugeas, J. P., Gu, Y., Leboeuf, C., Bouchtaoui, M. E. et al. (2019). High expression of apoptosis protein (Api-5) in chemoresistant triple-negative breast cancers: An innovative target. Oncotarget*,* 10 *(*61*),* 6577–6588. 10.18632/oncotarget.27312; 31762939 PMC6859922

[95] Cho, H., Chung, J. Y., Song, K. H., Noh, K. H., Kim, B. W. et al. (2014). Apoptosis inhibitor-5 overexpression is associated with tumor progression and poor prognosis in patients with cervical cancer. BMC Cancer*,* 14 *(*1*),* 545. 10.1186/1471-2407-14-545; 25070070 PMC4125689

[96] Chen, Z., Mai, Q., Wang, Q., Gou, Q., Shi, F. et al. (2022). CircPOLR2A promotes proliferation and impedes apoptosis of glioblastoma multiforme cells by up-regulating POU3F2 to facilitate SOX9 transcription. Neuroscience*,* 503*,* 118–130. 10.1016/j.neuroscience.2022.03.035; 35398178

[97] Cui, T., Bell, E. H., McElroy, J., Liu, K., Sebastian, E. et al. (2021). A novel miR-146a-POU3F2/SMARCA5 pathway regulates stemness and therapeutic response in glioblastoma. Molecular Cancer Research*,* 19*,* 48–60. 10.1158/1541-7786.MCR-20-0353; 32973101 PMC12685269

[98] Xia, H., Liu, B., Shen, N., Xue, J., Chen, S. et al. (2022). circRNA-0002109 promotes glioma malignant progression via modulating the miR-129-5P/EMP2 axis. Molecular Therapy-Nucleic Acids*,* 27*,* 1–15. 10.1016/j.omtn.2021.11.011; 34938603 PMC8646083

[99] Dillard, C., Kiyohara, M., Mah, V., McDermott, S. P., Bazzoun, D. et al. (2020). EMP2 is a novel regulator of stemness in breast cancer cells. Molecular Cancer Therapeutics*,* 19*,* 1682–1695. 10.1158/1535-7163.MCT-19-0850; 32451329 PMC7415657

[100] Wei, Y., Lu, C., Zhou, P., Zhao, L., Lyu, X. et al. (2021). EIF4A3-induced circular RNA ASAP1 promotes tumorigenesis and temozolomide resistance of glioblastoma via NRAS/MEK1/ERK1-2 signaling. Neuro-Oncology*,* 23 *(*4*),* 611–624. 10.1093/neuonc/noaa214; 32926734 PMC8041353

[101] Wesolowski, J. R., Rajdev, P., Mukherji, S. K. (2010). Temozolomide (Temodar). American Journal of Neuroradiology*,* 31 *(*8*),* 1383–1384. 10.3174/ajnr.A2170; 20538821 PMC7966084

[102] Chan, C. C., Dostie, J., Diem, M. D., Feng, W., Mann, M. et al. (2004). eIF4A3 is a novel component of the exon junction complex. RNA*,* 10 *(*2*),* 200–209. 10.1261/rna.5230104; 14730019 PMC1370532

[103] Liu, J., Song, S., Lin, S., Zhang, M., Du, Y. et al. (2019). Circ-SERPINE2 promotes the development of gastric carcinoma by sponging miR-375 and modulating YWHAZ. Cell Proliferation*,* 52 *(*4*),* e12648. 10.1111/cpr.12648; 31199037 PMC6668981

[104] Li, D., Li, L., Chen, X., Yang, W., Cao, Y. (2021). Circular RNA SERPINE2 promotes development of glioblastoma by regulating the miR-361-3p/miR-324-5p/BCL2 signaling pathway. Molecular Therapy-Oncolytics*,* 22*,* 483–494. 10.1016/j.omto.2021.07.010; 34553034 PMC8433060

[105] Liu, L., Xiao, S., Wang, Y., Zhu, Z., Cao, Y. et al. (2022). Identification of a novel circular RNA circZNF652/miR-486-5p/SERPINE1 signaling cascade that regulates cancer aggressiveness in glioblastoma (GBM). Bioengineered*,* 13 *(*1*),* 1411–1423. 10.1080/21655979.2021.2018096; 35258403 PMC8805984

[106] Zhang, Q., Lei, L., Jing, D. (2020). Knockdown of SERPINE1 reverses resistance of triple‐negative breast cancer to paclitaxel via suppression of VEGFA. Oncology Reports*,* 44*,* 1875–1884. 10.3892/or.2020.7770; 33000256 PMC7551184

[107] Wu, Y., Xie, Z., Chen, J., Chen, J., Ni, W. et al. (2019). Circular RNA circTADA2A promotes osteosarcoma progression and metastasis by sponging miR-203a-3p and regulating CREB3 expression. Molecular Cancer*,* 18 *(*1*),* 73. 10.1186/s12943-019-1007-1; 30940151 PMC6444890

[108] Zhang, Q., Wang, J. Y., Zhou, S. Y., Yang, S. J., Zhong, S. L. (2019). Circular RNA expression in pancreatic ductal adenocarcinoma. Oncology Letters*,* 18*,* 2923–2930. 10.3892/ol.2019.10624; 31452773 PMC6676441

[109] Wang, L., Tong, X., Zhou, Z., Wang, S., Lei, Z. et al. (2018). Circular RNA hsa_circ_0008305 (circPTK2) inhibits TGF-beta-induced epithelial-mesenchymal transition and metastasis by controlling TIF1γ in non-small cell lung cancer. Molecular Cancer*,* 17 *(*1*),* 140. 10.1186/s12943-018-0889-7; 30261900 PMC6161470

[110] Yang, H., Li, X., Meng, Q., Sun, H., Wu, S. et al. (2020). CircPTK2 (hsa_circ_0005273) as a novel therapeutic target for metastatic colorectal cancer. Molecular Cancer*,* 19 *(*1*),* 13. 10.1186/s12943-020-1139-3; 31973707 PMC6977296

[111] Chen, W., Wang, N., Lian, M. (2021). CircRNA circPTK2 might suppress cancer cell invasion and migration of glioblastoma by inhibiting miR-23a maturation. Neuropsychiatric Disease and Treatment*,* 17*,* 2767–2774. 10.2147/NDT.S297108; 34456566 PMC8387247

[112] Yang, X., Liu, Y., Zhou, X., Chen, K., Xu, J. et al. (2022). Circular RNA 0010117 promotes aggressive glioblastoma behavior by regulating the miRNA-6779-5p/SPEN axis. Translational Oncology*,* 25 *(*6*),* 101515. 10.1016/j.tranon.2022.101515; 36087384 PMC9468456

[113] Kong, X., Xu, R., Wang, W., Zeng, M., Li, Y. et al. (2021). CircularLRRC7 is a potential tumor suppressor associated with miR-1281 and PDXP expression in glioblastoma. Frontiers in Molecular Biosciences*,* 8*,* 743417. 10.3389/fmolb.2021.743417; 34912844 PMC8667166

[114] Schulze, M., Hutterer, M., Sabo, A., Hoja, S., Lorenz, J. et al. (2018). Chronophin regulates active vitamin B6 levels and transcriptomic features of glioblastoma cell lines cultured under non-adherent, serum-free conditions. BMC Cancer*,* 18 *(*1*),* 524. 10.1186/s12885-018-4440-4; 29724193 PMC5934884

[115] Gao, X., Xia, X., Li, F., Zhang, M., Zhou, H. et al. (2021). Circular RNA-encoded oncogenic E-cadherin variant promotes glioblastoma tumorigenicity through activation of EGFR-STAT3 signalling. Nature Cell Biology*,* 23 *(*3*),* 278–291. 10.1038/s41556-021-00639-4; 33664496

[116] Filbin, M. G., Dabral, S. K., Pazyra-Murphy, M. F., Ramkissoon, S., Kung, A. L. et al. (2013). Coordinate activation of Shh and PI3K signaling in PTEN-deficient glioblastoma: New therapeutic opportunities. Nature Medicine*,* 19 *(*11*),* 1518–1523. 10.1038/nm.3328; 24076665 PMC3923315

[117] Kool, M., Jones, D. T., Jager, N., Northcott, P. A., Pugh, T. J. et al. (2014). Genome sequencing of SHH medulloblastoma predicts genotype-related response to smoothened inhibition. Cancer Cell*,* 25 *(*3*),* 393–405. 10.1016/j.ccr.2014.02.004; 24651015 PMC4493053

[118] Xie, J. (2008). Hedgehog signaling pathway: Development of antagonists for cancer therapy. Current Oncology Reports*,* 10 *(*2*),* 107–113. 10.1007/s11912-008-0018-7; 18377823

[119] Wu, X. J., Xiao, S. H., Zhang, M. L., Yang, L. X., Zhong, J. et al. (2021). A novel protein encoded by circular SMO RNA is essential for Hedgehog signaling activation and glioblastoma tumorigenicity. Genome Biology*,* 22*,* 33. 10.1186/s13059-020-02250-6; 33446260 PMC7807754

[120] Zhang, M., Huang, N., Yang, X., Luo, J., Yan, S. et al. (2018). A novel protein encoded by the circular form of the SHPRH gene suppresses glioma tumorigenesis. Oncogene*,* 37 *(*13*),* 1805–1814. 10.1038/s41388-017-0019-9; 29343848

[121] Unk, I., Hajdu, I., Fatyol, K., Szakal, B., Blastyak, A. et al. (2006). Human SHPRH is a ubiquitin ligase for Mms2-Ubc13-dependent polyubiquitylation of proliferating cell nuclear antigen. Proceedings of the National Academy of Sciences of the United States of America*,* 103 *(*48*),* 18107–18112. 10.1073/pnas.0608595103; 17108083 PMC1838714

[122] Song, J., Zheng, J., Liu, X., Dong, W., Yang, C. et al. (2022). A novel protein encoded by ZCRB1-induced circHEATR5B suppresses aerobic glycolysis of GBM through phosphorylation of JMJD5. Journal of Experimental & Clinical Cancer Research*,* 41 *(*1*),* 171. 10.1186/s13046-022-02374-6; 35538499 PMC9086421

[123] Wang, H. J., Hsieh, Y. J., Cheng, W. C., Lin, C. P., Lin, Y. S. et al. (2014). JMJD5 regulates PKM2 nuclear translocation and reprograms HIF-1α-mediated glucose metabolism. Proceedings of the National Academy of Sciences of the United States of America*,* 111 *(*1*),* 279–284. 10.1073/pnas.1311249111; 24344305 PMC3890888

[124] Xia, X., Li, X., Li, F., Wu, X., Zhang, M. et al. (2019). A novel tumor suppressor protein encoded by circular AKT3 RNA inhibits glioblastoma tumorigenicity by competing with active phosphoinositide-dependent Kinase-1. Molecular Cancer*,* 18 *(*1*),* 131. 10.1186/s12943-019-1056-5; 31470874 PMC6716823

[125] Chin, Y. R., Yuan, X., Balk, S. P., Toker, A. (2014). PTEN-deficient tumors depend on AKT2 for maintenance and survival. Cancer Discovery*,* 4*,* 942–955. 10.1158/2159-8290.Cd-13-0873; 24838891 PMC4125464

[126] Zhao, H. F., Wang, J., Shao, W., Wu, C. P., Chen, Z. P. et al. (2017). Recent advances in the use of PI3K inhibitors for glioblastoma multiforme: Current preclinical and clinical development. Molecular Cancer*,* 16*,* 100. 10.1186/s12943-017-0670-3; 28592260 PMC5463420

[127] Yuan, F., Sun, Q., Xu, Y., Zhang, H., Deng, G. et al. (2021). Hsa_circ_0072309 inhibits proliferation and invasion of glioblastoma. Pathology Research and Practice*,* 222*,* 153433. 10.1016/j.prp.2021.153433; 33862563

[128] Cordonnier, T., Bishop, J. L., Shiota, M., Nip, K. M., Thaper, D. et al. (2015). Hsp27 regulates EGF/β-catenin mediated epithelial to mesenchymal transition in prostate cancer. International Journal of Cancer*,* 136 *(*6*),* E496–E507. 10.1002/ijc.29122; 25130271

[129] Liu, Y., Ao, X., Zhou, X. H., Du, C. C., Kuang, S. X. (2022). The regulation of PBXs and their emerging role in cancer. Journal of Cellular and Molecular Medicine*,* 26 *(*5*),* 1363–1379. 10.1111/jcmm.17196; 35068042 PMC8899182

[130] Liu, Y., Wang, Y., Li, X., Jia, Y., Wang, J. et al. (2022). FOXO3a in cancer drug resistance. Cancer Letters*,* 540*,* 215724. 10.1016/j.canlet.2022.215724; 35545128

[131] Stella, M., Falzone, L., Caponnetto, A., Gattuso, G., Barbagallo, C. et al. (2021). Serum extracellular vesicle-derived circHIPK3 and circSMARCA5 are two novel diagnostic biomarkers for glioblastoma multiforme. Pharmaceuticals*,* 14 *(*7*),* 618. 10.3390/ph14070618; 34198978 PMC8308516

[132] Chen, A., Zhong, L., Ju, K., Lu, T., Lv, J. et al. (2020). Plasmatic circRNA predicting the occurrence of human glioblastoma. Cancer Management and Research*,* 12*,* 2917–2923. 10.2147/CMAR.S248621; 32425605 PMC7196774

[133] Xia, D., Gu, X. (2021). Plasmatic exosome-derived circRNAs panel act as fingerprint for glioblastoma. Aging*,* 13 *(*15*),* 19575–19586. 10.18632/aging.203368; 34385405 PMC8386567

[134] Liu, Y., Ao, X., Wang, Y., Li, X., Wang, J. (2022). Long non-coding RNA in gastric cancer: Mechanisms and clinical implications for drug resistance. Frontiers in Oncology*,* 12*,* 841411. 10.3389/fonc.2022.841411; 35155266 PMC8831387

[135] Chen, F., Guo, L., Di, J., Li, M., Dong, D. et al. (2021). Circular RNA ubiquitin-associated protein 2 enhances autophagy and promotes colorectal cancer progression and metastasis via miR-582-5p/FOXO1 signaling. Journal of Genetics and Genomics*,* 48 *(*12*),* 1091–1103. 10.1016/j.jgg.2021.07.017; 34416339

[136] Xiong, H., Yu, J., Jia, G., Su, Y., Zhang, J. et al. (2021). Emerging roles of circUBAP2 targeting miR-370-3p in proliferation, apoptosis, and invasion of papillary thyroid cancer cells. Human Cell*,* 34 *(*6*),* 1866–1877. 10.1007/s13577-021-00585-1; 34346032

[137] Xu, Z. Q., Yang, M. G., Liu, H. J., Su, C. Q. (2018). Circular RNA hsa_circ_0003221 (circPTK2) promotes the proliferation and migration of bladder cancer cells. Journal of Cellular Biochemistry*,* 119 *(*4*),* 3317–3325. 10.1002/jcb.26492; 29125888

